# The Safety and Effectiveness of Intravenous Contrast-Enhanced Sonography in Chinese Children—A Single Center and Prospective Study in China

**DOI:** 10.3389/fphar.2019.01447

**Published:** 2019-12-05

**Authors:** Muyi Mao, Bei Xia, Weiling Chen, Xiaojie Gao, Jun Yang, Shoulin Li, Bin Wang, Huirong Mai, Sixi Liu, Feiqiu Wen, Yungen Gan, Jianming Song, Hong Wei, Weiguo Yang, Yuhui Wu, Shufang Yang, Wei Yu, Hongkui Yu, Shumin Fan, Hongwei Tao, Xia Feng, Zhou Lin, Lei Liu

**Affiliations:** ^1^Department of Ultrasound, Shenzhen Children’s Hospital, Shenzhen, China; ^2^Department of Nephrology and Hematology, Shenzhen Children’s Hospital, Shenzhen, China; ^3^Department of Rheumatology and Immunology, Shenzhen Children’s Hospital, Shenzhen, China; ^4^Department of Urinary Surgery, Shenzhen Children’s Hospital, Shenzhen, China; ^5^Department of General Surgery, Shenzhen Children’s Hospital, Shenzhen, China; ^6^Department of Hematology and Oncology, Shenzhen Children’s Hospital, Shenzhen, China; ^7^Radiology Department, Shenzhen Children’s Hospital, Shenzhen, China; ^8^Department of Pathology, Shenzhen Children’s Hospital, Shenzhen, China; ^9^Drug Clinical Trial Center, Shenzhen Children’s Hospital, Shenzhen, China; ^10^Intensive Care Unit, Shenzhen Children’s Hospital, Shenzhen, China

**Keywords:** ultrasound, contrast-enhanced ultrasound, safety, effectiveness, child

## Abstract

**Background and Objective:** Intravenous contrast-enhanced ultrasound (CEUS), using the second-generation ultrasound contrast agent SonoVue^®^, has been widely used in adults. In 2016, it was approved for pediatric applications by the American Food and Drug Administration (FDA). However, it has not been approved by the Chinese Food and Drug Administration (CFDA). The objective of the study was to evaluate the safety and effectiveness of CEUS in children prospectively at a single center in China.

**Methods:** A total of 312 cases of Chinese children were enrolled in clinical trials. Contrast agent was given intravenously with two different doses, including 2.4 ml/time and 0.03 ml/kg. All CEUS was performed for evaluating adverse effect and the diagnostic accuracy compared with the pathology and enhanced CT.

**Results:** All 312 subjects underwent CEUS successfully. The dose of contrast agent for CEUS was 2.4 ml between November 2015 and June 2016, which was modified to 0.03 ml/kg between July 2016 and April 2019, according to the recommendation of the FDA. With the two different doses of the contrast agent, the heart rate, respiration rate, oxygen saturation, and blood pressure of the participants had no statistically significant difference (*P* > 0.05) before and after administration. The blood pressure had been significantly decreased in participants who received combined anesthetic administration. Following 600 intravenous injections of the CEUS, there were three cases of transient rash and three cases of hypotension (n = 6, 1.92%). The six recovered quickly after receiving intravenous methylprednisolone and epinephrine. Most of the studies were performed for evaluating renal microcirculation and assisting renal biopsy (192/312 [61.5%]), which together had a 98.9% effectiveness in the identification of pathology in the specimens. Some studies were conducted to identify a mass in the liver, retroperitoneum, abdominal cavity, kidneys, testicles, thyroid, and so on (99/312 [31.4%]), which had a 97.6% accuracy. The other studies were conducted to identify trauma, vascular malformation, infection, hemorrhage, and so on (21/312 [6.73%]), which had a similar accuracy to enhanced CT.

**Conclusion:** The adverse effects of CEUS in children are similar to that in adults. The results indicate that it is safe to use SonoVue^®^ for CEUS in pediatric patients.

## Introduction

Contrast-enhanced ultrasound (CEUS) has been widely used in adults. In 2016, it was approved for intravenous use to diagnose liver lesions and intravesical use in children, basing on the big data from studies about safety and feasibility with SonoVue^®^/Lumason^®^ by the FDA. However, it lacked relevant studies in China. The study was to evaluate the safety and feasibility of intravenous CEUS with SonoVue^®^ in children under 18 years of age.

## Patients And Methods

### Patients

The study was performed in Shenzhen Children’s Hospital between November 2015 and April 2019. A total of 312 Chinese children were enrolled in clinical trials and 600 intravenous injections were performed. All subjects met the inclusion and exclusion criteria as follows.

Inclusion criteria

1. Inpatients or outpatients from birth to 18 years old.
2. Performance of CEUS was needed for diagnosis.
3. Bedside CEUS for diagnosis instead of CT and MR.
4. Agreed to be enrolled into the trial and signed the informed consent.

Exclusion criteria

1. Allergy to sulfur hexafluoride (SF6) or other components of SonoVue^®^.
2. Having an allergic history of protein or egg.
3. Congenital heart disease with right-to-left shunt, severe pulmonary hypertension, or uncontrolled hypertension.
4. Severe respiratory failure.
5. Without informed consent.

The prospective study with SonoVue^®^ was approved by the Chinese Ethics Committee (No: 2016[003], ChiCTR-ONh-16009236).

### The Protocol of CEUS

All US examinations, including baseline US and CEUS, were performed with 4.5–9.0-MHz C2-9 convex array probes and 5.0–9.0-MHz 9L linear probes in the US system (LogiQ E9 Ultrasound System; GE Healthcare, Milwaukee, WI). And the mechanical index (MI) during the CEUS examination was lower than 0.2.

The preventive measures for allergy were taken in trials. We weighed all subjects and calculated the doses of anti-allergic medications, including 0.01 mg/kg of 0.1% adrenaline (the maximal dose 0.3 mg) and 1∼2 mg/kg of methylprednisolone, and prepared a rescue equipment with the medications as a routine.

The contrast agent SonoVue^®^ was administered in two different ways: 2.4 ml/time of SonoVue^®^ before June 2016, while 0.03 ml/kg/time between July 2016 and April 2019, according to the recommendation of FDA. The contrast agents were bolus injected through peripheral veins within 3s, and then the irrigation of the catheters with 0.9% of sodium chloride solutions, keeping a velocity of about 2 ml/s, were performed. All subjects underwent different times of CEUS examinations, including 34 cases undergoing one CEUS, 270 cases undergoing two continuous CEUS, 6 cases undergoing three continuous CEUS, and 1 case undergoing four times.

The CEUS examinations and the intravenous bolus injections with SonoVue^®^ were started simultaneously. The views and images of CEUS were acquired and stored until the microbubbles were cleared up, and the wash-in time, time to peak, and wash-out time were recorded in the whole examinations. The heart rate, respiratory rate, oxygen saturation, and blood pressure were observed and recorded respectively at three different times: before administration, immediately after administration, and 15 min after administration. As for awake participants, we inquired and made a record of any discomfort, such as pain, nausea, vomiting, and so on, besides observing the appearance and the rash. The follow-ups, which contained vital sign observation and rash management, were performed within 24 h before CEUS, 24 h after CEUS, and 72 h after CEUS. If it was necessary, for some subjects having a need for more than one intravenous CEUS, especially for the subjects with a diagnostic purpose, the next injections of SonoVue^®^ were performed at least 20 min later.

The images were evaluated for the following: ①hepatic lesions, such as vascular malformation, abscess, hyperplasia, and so on; ②infarction and necrosis in spleen, kidney, and other solid organs; ③distinction between benign and malignant lesions; ④perfusion and blood supply of the mass and guiding the puncture; ⑤active hemorrhage of the site after puncture; ⑥complications after puncture, especially the vascular complications; and ⑦ distinguishing the hematoma from mass.

### Statistical Analysis

The SPSS software (SPSS, version 21.0, IBM) was employed for statistical analysis. Means and standard deviations were used to summarize continuous data for descriptive statistics purposes. The patients’ characteristics were evaluated by using *t*-tests for continuous variables, while descriptions were made for categorical data. The significance was set at a *p*-value < 0.05.

## Results

### General Information and Vital Signs

Among 312 participants, 179 were male and 133 were female. The median age was 7.08 years old (from 5 days to 14.5 years) and the median weight was 23 kg (3.7–65.0 kg). 287 cases had complete records of vital sign, including heart rate (HR), respiration rate (RR), oxygen saturation (SO_2_), systolic blood pressure (SBP), and diastolic blood pressure (DBP), before and after administration. Ten cases of the records were shown as follows (**Table 1**).

**Table 1:** Ten cases of the records before and after administration.

Case number	Gender	Age	Weight	Condition	Before administration					After administration				
					HR (/min)	RR (/min)	SO_2_ (%)	SBP (mmHg)	DBP (mmHg)	HR (/min)	RR (/min)	SO_2_ (%)	SBP (mmHg)	DBP (mmHg)
Case 1	male	3y3m	15.3	combined anesthesia	125	30	100	96	53	117	30	100	95	49
Case 2	male	3y1m	15	combined anesthesia	116	19	98	88	51	122	20	97	84	52
Case 3	male	9y3m	32	combined anesthesia	111	30	98	87	51	113	30	98	88	53
Case 4	female	13y6m	46.3	combined anesthesia	111	25	98	102	71	114	24	99	107	72
Case 5	male	10m	10.6	combined anesthesia	108	24	100	132	60	105	24	100	105	61
Case 6	female	5y8m	17	awake	116	18	97	80	42	115	20	97	79	40
Case 7	male	14y4m	57	awake	79	28	100	116	72	78	28	100	121	67
Case 8	female	5y6m	19	awake	110	30	99	98	72	112	30	99	98	68
Case 9	male	4m	7.2	sedation	128	20	99	125	78	134	20	99	125	78
Case 10	male	5y	18	coma	78	30	100	136	79	80	30	100	135	80

### The Vital Signs Before and After Administration

All the 312 participants underwent CEUS successfully. They included: 228 cases in combined anesthesia, 63 cases in awake state, 18 cases in sedation with chloral hydrate, 3 cases in coma. Comparing the vital signs after administration with that before administration in both combined anesthetic and awake participants, there was a statistically significant difference (*P* < 0.05) in the combined anesthetic participants (**Tables 2** and **3**).

**Table 2:** The vital signs of the combined anesthetic participants.

Subjects (n = 228)	Heart rate (/min)	Respiration rate (/min)	Oxygen saturation (%)	Systolic blood pressure (mmHg)	Diastolic blood pressure (mmHg)
Before administration	108.79 ± 18.33	22.83 ± 3.84	98.80 ± 1.82	101.78 ± 15.24	60.03 ± 13.23
After administration	108.16 ± 17.44	22.80 ± 3.68	98.95 ± 1.36	99.69 ± 14.87	57.92 ± 13.24
*t*	0.834	0.329	−1.388	3.829	5.086
*P*	0.408	0.743	0.167	<0.001*	<0.001*

*The significance was set at a p-value < 0.05 in the study.

**Table 3:** The vital signs of the awake participants.

Subjects (n = 63)	Heart rate (/min)	Respiration rate (/min)	Oxygen saturation (%)	Systolic blood pressure (mmHg)	Diastolic blood pressure (mmHg)
Before administration	102.14 ± 22.00	23.68 ± 4.56	99.43 ± 1.17	104.94 ± 15.92	65.05 ± 10.81
After administration	100.27 ± 21.37	24.11 ± 5.08	99.48 ± 1.06	104.79 ± 14.04	65.68 ± 11.28
*t*	1.699	−1.989	−0.554	0.148	−0.704
*P*	0.094	0.051	0.582	0.883	0.484

The vital signs of 18 cases in sedation with chloral hydrate showed as follows: no statistically significant difference (*P* < 0.05) was found (**Table 4**).

**Table 4:** The vital signs of the participants in sedation with chloral hydrate.

Subjects (n = 18)	Heart rate (/min)	Respiration rate (/min)	Oxygen saturation (%)	Systolic blood pressure (mmHg)	Diastolic blood pressure (mmHg)
Before administration	117.44 ± 18.64	23.33 ± 4.14	99.39 ± 0.61	90.78 ± 20.14	56.22 ± 17.20
After administration	117.89 ± 18.55	24.61 ± 6.04	99.56 ± 0.62	91.22 ± 20.57	58.94 ± 16.00
*t*	−0.266	−1.668	−1.374	−0.389	−1.690
*P*	0.793	0.114	0.187	0.702	0.109

In comatose participants, the vital signs after administration were not statistically significant different (*P* < 0.05) from the corresponding signs before administration: heart rate (102.67 ± 26.10 vs. 105.33 ± 26.03) beats/min,*t* = −4.000, *P* = 0.057; respiratory rate (30.00 ± 2.00 vs. 30.33 ± 1.53 breaths/min,*t* = −1.000, *P* = 0.423; oxygen saturation (95.33 ± 5.03 vs. 96.67 ± 5.77)%,*t* = 1.000, *P* = 0.423); systolic blood pressure (107.67 ± 26.69 vs. 109.00 ± 26.00 mmHg,*t* = −0.718, *P* = 0.547); and diastolic blood pressure (58.00 ± 18.52 vs. 57.67 ± 19.50 mmHg,*t* = 0.378, *P* = 0.742).

### Adverse Effects

**Figure 1:**
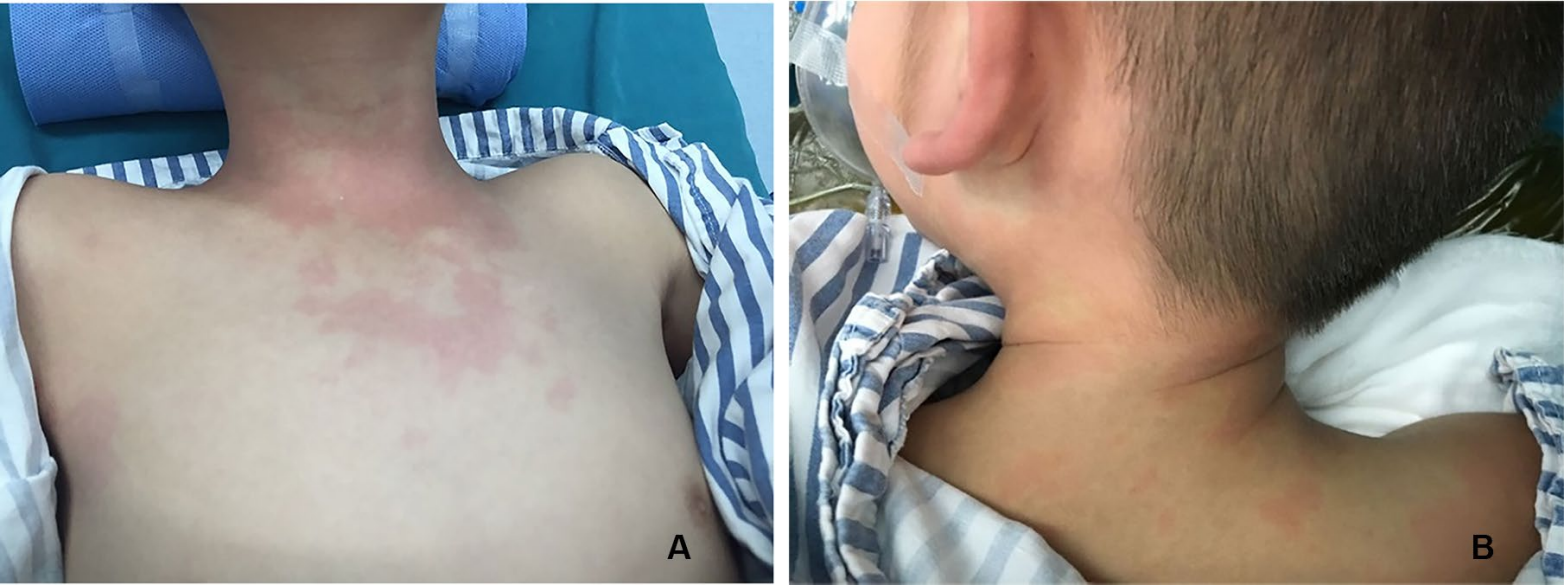
Rash in chest **(A)** and neck **(B)** after administration of contrast agent.

There were three cases of rash (**Figure 1**) and three cases of hypotension (**Table 5**).

**Table 5:** The allergies and the managements.

	Gender	Age	Clinical diagnosis	Drug batch number	Time of allergy	Allergic performance	Management	Outcome
Case1	Male	5Y8M	Solitary hematuria	17A011A	The first CEUS	Chest rash (**Figure 1A**) with stable condition	Intravenous injection of methylprednisolone 20 mg	Complete relief
Case 2	Male	7Y7M	Henoch-Schonlein purpura nephritis	17A011A	The second CEUS	Rash in posterior neck, facial, and back	Intravenous injection of methylprednisolone 40 mg	Complete relief
Case 3	Female	7Y11M	Nephrotic syndrome	17A023A	The second CEUS	Flushed face, and rash in posterior neck, facial, and back	Intravenous injection of methylprednisolone 40 mg	Complete relief
Case 4	Male	13Y3M	Inflammatory myofibroblastic tumor	17A023A	The first CEUS	Rash in face, chest, and abdominal, a high heart rate (148 bpm), hypotension (66/34 mmHg)	Intravenous injection of dexamethasone 5 mg, methylprednisolone 40 mg, calcium gluconate 10 ml, adrenaline ((0.02 + 0.03+0.05) mg, 0.05 mg/kg*min)	Complete relief
Case 5	Male	6Y3M	Henoch-Schonlein purpura nephritis	17A038A	The first CEUS	Continuous cough, hypotension (53/17 mmHg)	Intravenous injection of methylprednisolone 40 mg	Complete relief
Case 6	Female	6Y3M	Nephrotic syndrome	17A052A	The first CEUS	Hypotension (78/47 mmHg)	Intravenous injection of methylprednisolone 40 mg	Complete relief

### CEUS and the Enhancement Modality

In our study, CEUS was performed for many organs, including liver, kidney, spleen, pancreas, thyroid, testicles, and so on. The arrival time (AT), time to peak (TTP), and the wash-out time were documented in **Table 6**.

**Table 6:** The arrival time, time to peak, and the wash-out time of liver and kidney.

	Arrival time (s)	Time to peak (s)	Wash-out time (s)
Liver (35 cases)	8.75 ± 2.06	26.25 ± 1.25	462.50 ± 2.33.24
Kidney (187 cases)	10.17 ± 3.83	24.41 ± 6.53	450.98 ± 164.18

With different doses, renal CEUS was performed and the wash-out time was recorded. It was showed below (**Table 7**).

**Table 7:** The wash-out time of renal CEUS with different doses of contrast agents.

Wash-out time (s)	
Dose 1	517.91 ± 138.78
Dose 2	386.38 ± 102.96
*t*	7.001
*P*	0.025

Dose 1: 2.4 ml/time; Dose 2: 0.03 ml/kg/time. And one case can’t acquire the wash-out time because of the severe adverse effect.

### The Accuracy and Feasibility of CEUS

Among 312 subjects, 287 cases were made the terminal diagnosis by biopsy, surgery, or clinical diagnostic standards, while 25 cases were available for follow-up. (**Table 8**)

**Table 8:** The clinical or pathologic diagnosis of the subjects.

Diffuse Renal Lesions (cases)	Mass (cases)	Others (cases)	Loss of Follow-ups (cases)
Minimal change disease (MCD) (60)	Hepatoblastoma (11)	Gastrointestinal hemorrhage (2)	Hepatic mass (8)
Purpura nephritis (44)	Hepatocellular carcinoma (3)	Postoperative hemorrhage (3)	Renal mass (3)
IgA nephropathy (32)	Neuroblastoma (15)	Subcutaneous hemorrhage (1)	Cervical mass (2)
lupus nephritis (LN) (19)	Lymphoma (12)	Trauma (2)	Testicular mass (1)
Mesangial proliferative glomerulonephritis (MsPGN) (8)	Teratoma (6)	Renal segmental infarction (1)	Thyroid nodules (1)
Thin basement membrane nephropathy(TBMN)(7)	Yolk sac tumor (3)	Hepatic vascular malformation (2)	Pelvic mass (1)
Focal segmental glomerular sclerosis (FSGS) (3)	Rhabdomyosarcoma (3)	Budd-Chiari syndrome (2)	Retroperitoneal mass (1)
Alport syndrome (2)	Nephroblastoma (2)	Venous thrombosis (2)	Diffuse renal lesions (8)
Membranous nephropathy (MN) (2)	Hemangioma (4)	Infection (4)	
Thrombotic microangiopathy (TMA) (1)	Langerhans cell histiocytosis (LCH) (2)	Fatty liver (1)	
Hepatitis B virus-associated membranous nephritis (HBV-MN) (1)	Inflammatory myofibroblastic tumor (IMT) (2)		
Endocapillary proliferative glomerulonephritis (EPGN) (1)	Malignant triton tumor (1)		
Crescentic glomerulonephritis (1)	Clear cell sarcoma (1)		
Interstitial nephritis (1)	Malignant rhabdoid tumor (MRT) (1)		
Sclerosing glomerulonephritis (1)	Pheochromocytoma (1)		
	Cystic nephroma (1)		
Failing to acquire the glomeruli (2)	Fibrosarcoma (1)		
	Malignant mixed germ cell tumor (1)		
	Neurofibroma (1)		
	Solid-pseudopapillary tumor (SPT) (1)		
	Benign lymph nodes (1)		
	Papillary thyroid carcinoma (PTC) (1)		
	Renal cyst (2)		
	Splenic cyst (1)		
	Small cell lung cancer (1)		
	Focal nodular hyperplasia (FNH) (1)		
	Glycogen storage disease (GSD) (1)		

**Figure 2:**
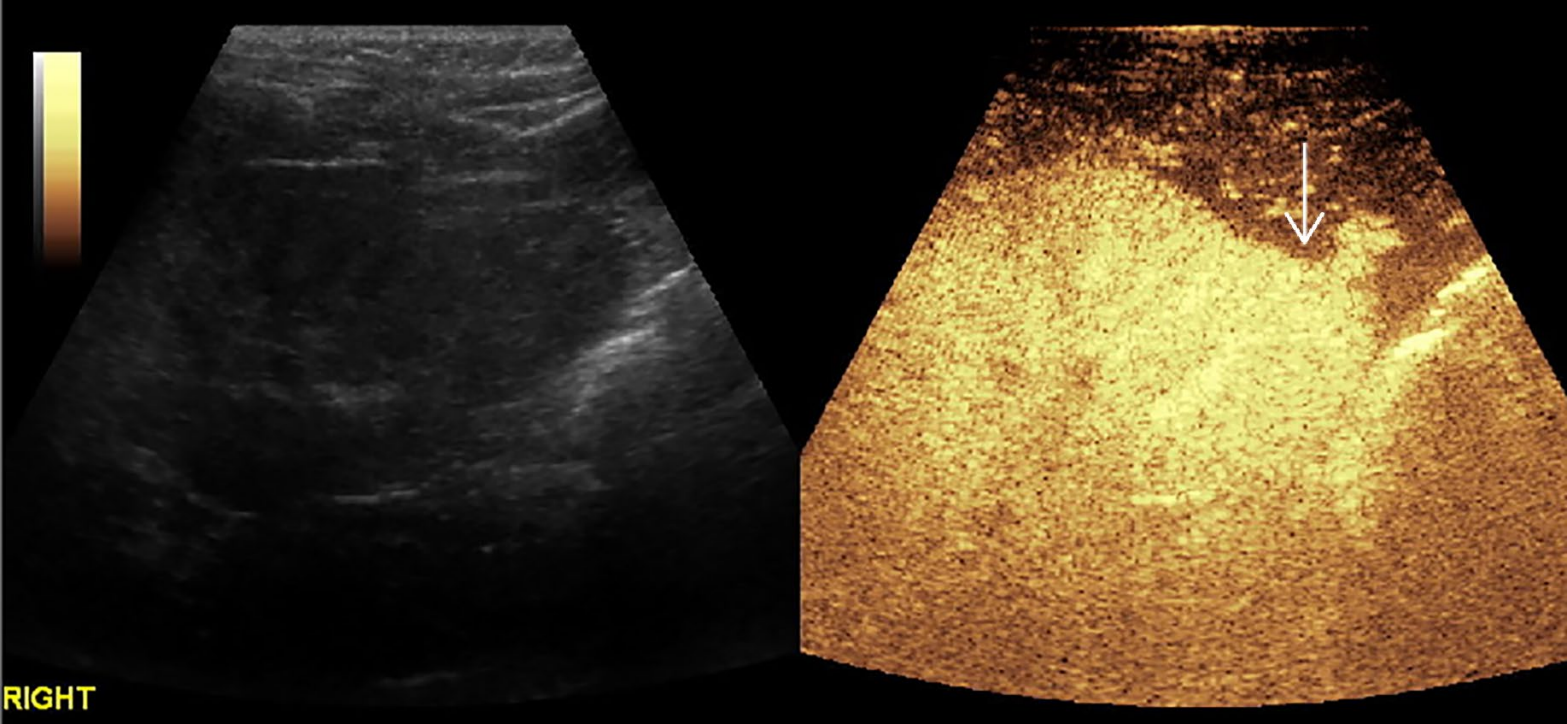
In a 5-year-old boy with IgA nephropathy, CEUS showed the microbubble exuded from kidney to abdominal cavity (arrow) after puncture.

**Figure 3:**
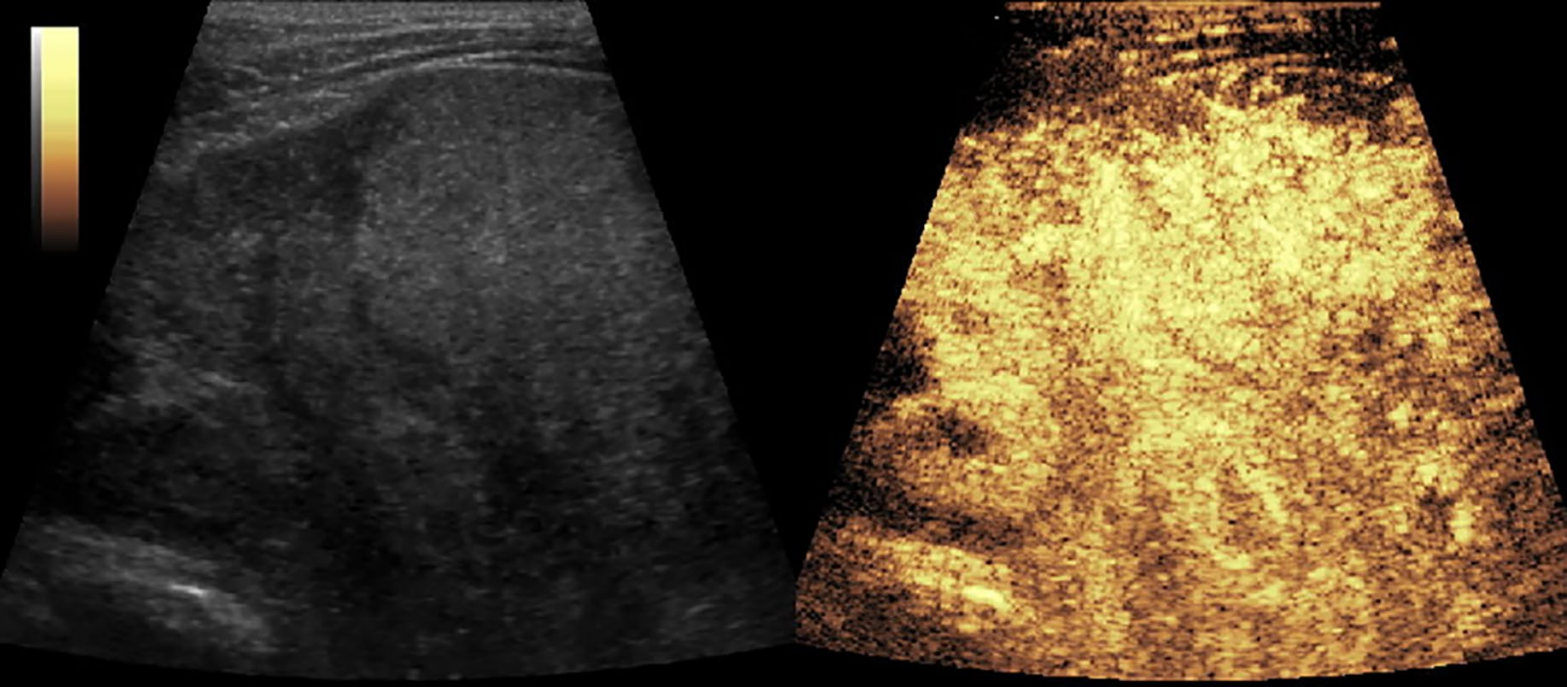
CEUS of hepatocellular carcinoma. The mass had a rapid enhancement and became heterogeneous.

**Figure 4:**
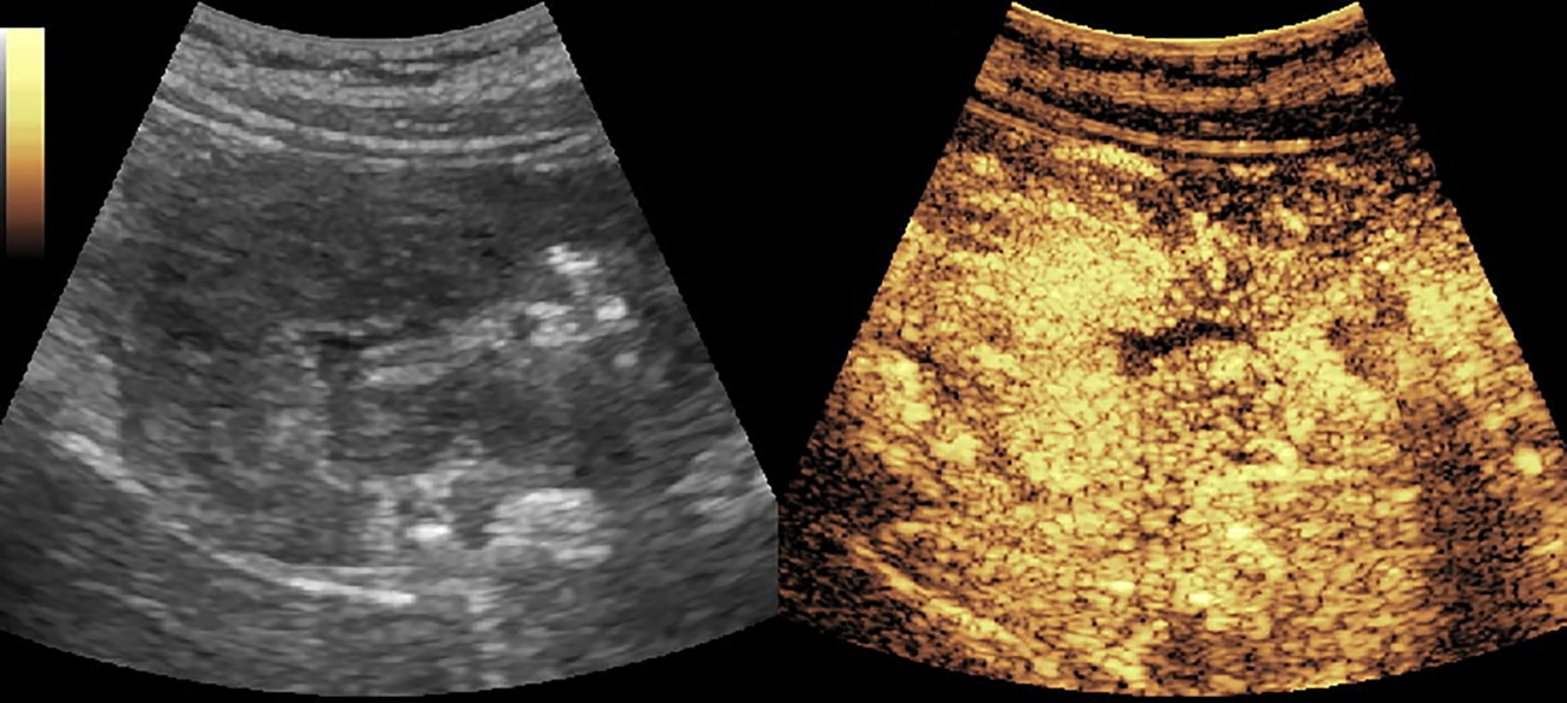
CEUS of inflammatory myofibroblastic tumor (IMT) showed a heterogeneous enhancement and rapid wash-out in the thickened gastric and intestinal wall.

**Figure 5:**
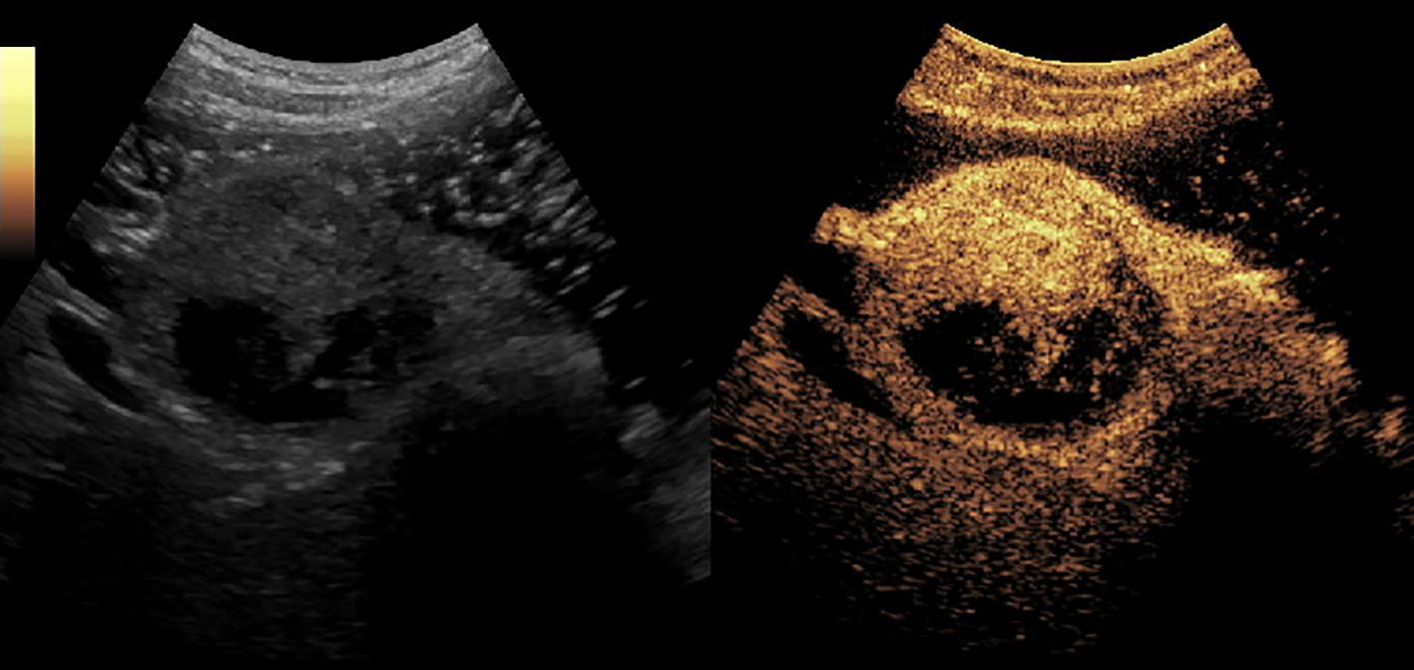
A solid-pseudopapillary tumor of pancreas (SPT) in the head of pancreas. It enhanced simultaneously with the normal pancreas parenchyma and had non-enhanced loculi inside it.

**Figure 6:**
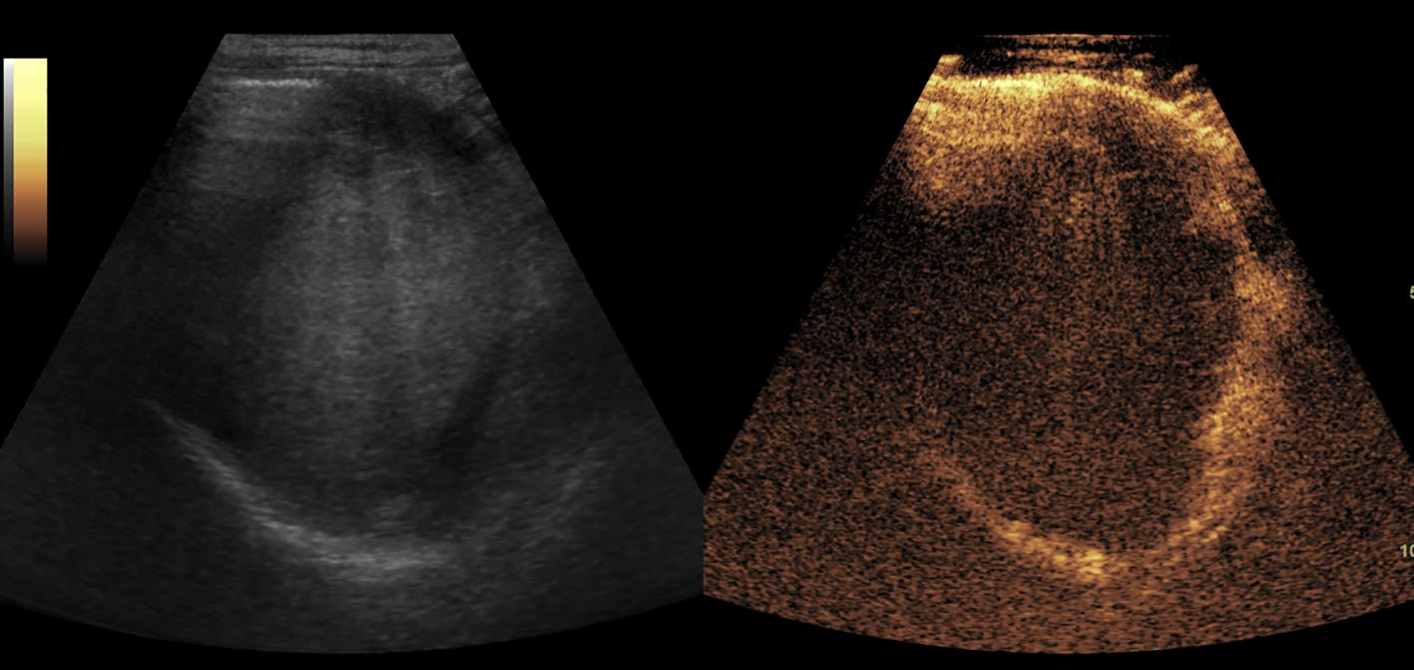
The blood clot in stomach. It showed as an isoechoic mass in baseline US and no enhancement in CEUS.

**Figure 7:**
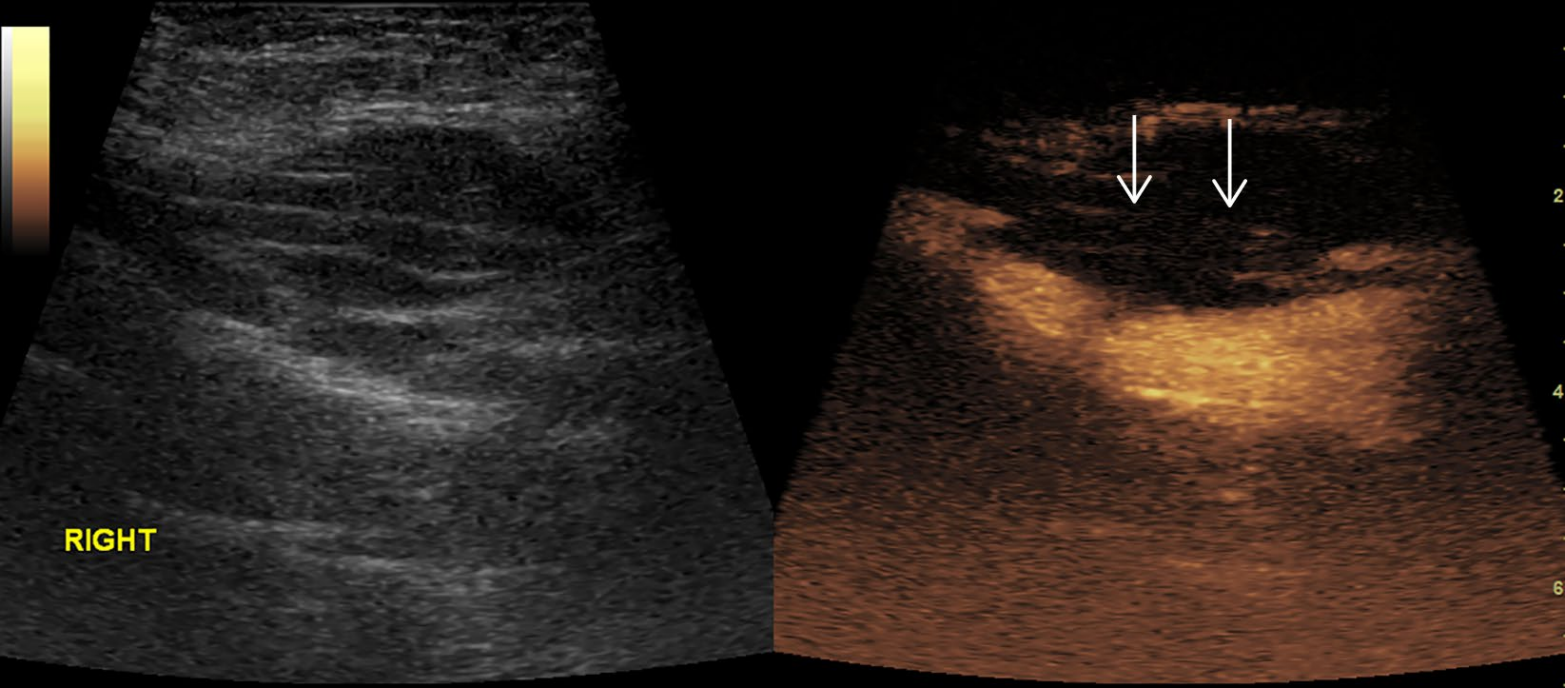
Thrombosis in superficial femoral artery. No enhancement in the proximal part (arrow) of right superficial femoral artery in CEUS.

**Figure 8:**
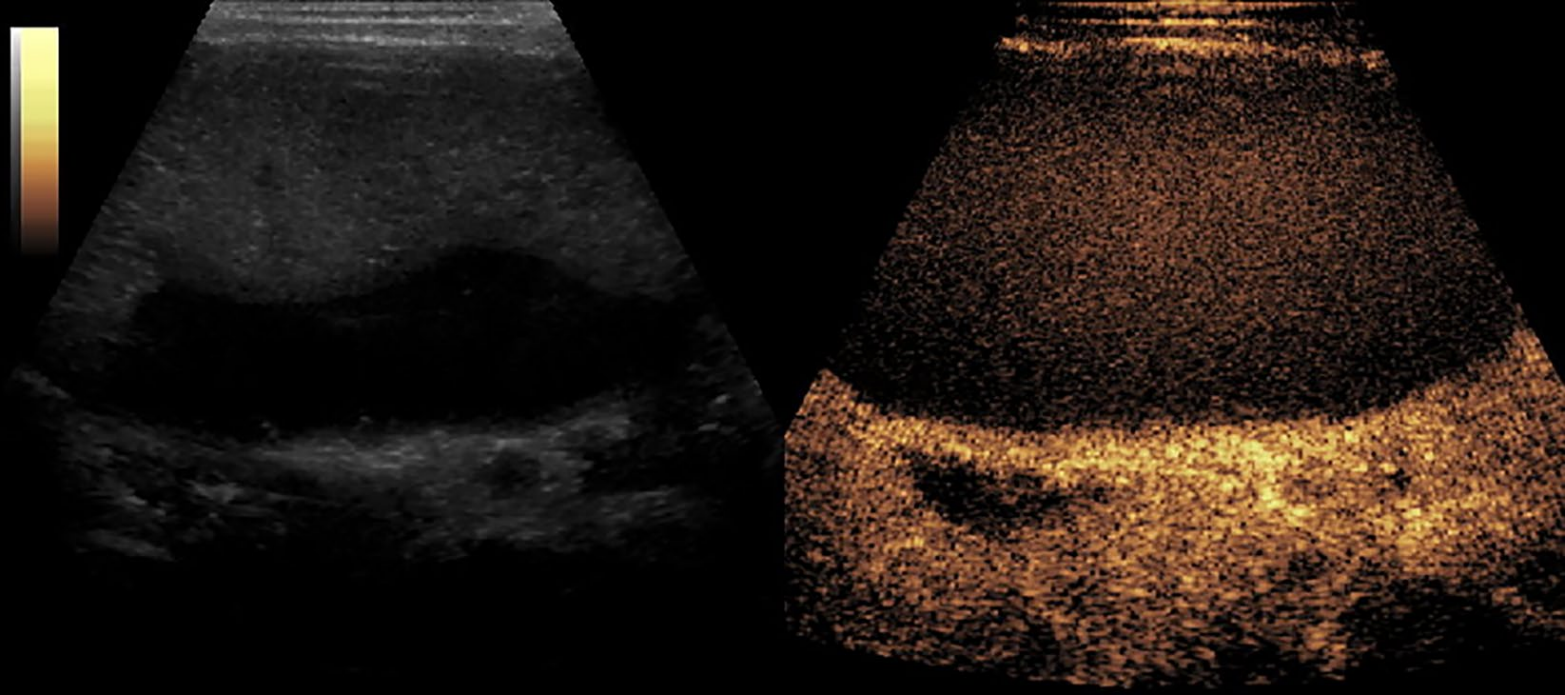
CEUS of Splenic necrosis. It showed as splenomegaly, non-enhancement in the spleen parenchyma.

**Figures 2**–**8** respectively showed the case of post-punctural active hemorrhage of kidney (**Figure 2**), hepatocellular carcinoma (**Figure 3**), inflammatory myofibroblastic tumor (IMT) (**Figure 4**), solid-pseudopapillary tumor of pancreas (SPT) (**Figure 5**), blood clot in stomach (**Figure 6**), thrombosis in superficial femoral artery (**Figure 7**), Bacillus Calmette-Guerin(BCG) vaccination disease with splenic necrosis (**Figure 8**), and renal segmental infarction.

Most of the studies were performed for evaluating renal microcirculation and assisting renal biopsy (192/312 [61.5%]), which had a 98.9% effectiveness in the identification of pathology in the specimens (**Table 9**).

**Table 9:** The effective rate of the pathological specimens with renal CEUS.

Cases for renal biopsy with renal CEUS	Cases with effective pathological specimen	Cases with ineffective pathological specimen	Effective rate
192	190	2 cases failing to acquire the glomeruli	98.9%

Some were used to identify the mass in almost the whole body, including 99 (31.4%) cases for the liver (31 cases), retroperitoneum (14 cases), abdominal cavity (14 cases), mediastinum (6 cases), kidney (10 cases), testicles (4 cases), thyroid (3 cases), pancreas (2 cases), spleen (3 cases), and so on. Except for loss to follow-up for 17 cases, it had a 97.6% accuracy as shown by the comparison of CEUS findings with pathology. The others were for identifying 21 (6.73%) cases for trauma, vascular malformation, infection, thrombosis, hemorrhage, infarction, and fatty liver, which had the same accuracy compared with enhanced CT (**Table 10**).

**Table 10:** The effective rate of the pathological specimens with renal CEUS.

Cases for identifying the mass with CEUS	Loss of following up	Cases for CEUS finding fitting to the pathology	Cases unfitting to the pathology	Accurate rate
99	17	80	2	97.6%

## Discussion

SonoVue^®^ has been widely used in adults in the recent years, and has been proven to be safe (Piscaglia and Bolondi, 2006). However, owing to lack of relevant guideline or support from clinical trials, it has had a slow approval for clinical use in pediatric cases. The intravenous CEUS in children was off-label use, although it was reported in multiple centers in Europe (Claudon et al., 2013; Sidhu et al., 2017). In April 2016, SonoVue^®^ was approved for non-cardiac use in pediatric patients by the FDA. In January 2017 it was approved for intravesical use, among pediatric cases. As for China, the pediatric application of SonoVue^®^ has not been approved by the CFDA due to the lack of relevant safety data from phase IV clinical trials.

Our study institute was qualified to conduct the clinical trial, due to the qualified staff, standard equipment, and the approval of the Ethics Committee.

Our results showed that CEUS was effective for the diagnosis of most diseases, especially those that were associated with the microcirculation. (Schreiber-Dietrich and Dietrich, 2012; Darge et al., 2013; Stenzel and Mentzel, 2014; Muyi and Bei, 2018)

CEUS in pediatric interventional therapy had its own advantage. In our study, 174 cases undergoing renal CEUS provided clinically useful results pertaining to evaluating the renal microcircular perfusion and the hemorrhage after puncture. This enabled the early identification of stanch bleeding in time. Forty-six (n = 46) cases for mass puncture evaded the necrosis and facilitated the acquisition of eligible tissue for pathology. After intravenous injection, microbubble became the maker of the vascular bed, which made the blood vessels and tissue perfusion images very clear due to the enhancement, in both the great vessels as well as the capillaries. The avascular, potential necrotic and liquefied areas could be recognized due to the expansion of the vision in the interventional operation and avoiding the damage of adjacent normal tissue after enhancement. Many structures were hard to recognize in B-mode US, but were exhibited in CEUS.

In our study, CEUS was performed for evaluating the post-traumatic kidney, renal infarction, splenic cyst, splenic necrosis, splenic hemangioma, hemorrhage in stomach and duodenum, arterial thrombosis, and mass in various organs, and had a high diagnostic accuracy rate compared with the result of surgery pathology and clinical follow-up. Besides, CEUS has been already used in organ transplantation. It was demonstrated that CEUS can help to access the vessels of target organs, the perfusion of transplanted organs, and the post-transplanted complications (Pschierer et al., 2015). CEUS can be used for evaluating the mass before and after radiation ablation (Xiaoer et al., 2016), including the mass size, the boundary, the location, and the therapeutic effect. In addition to the intravenous applications, CEUS can be used for urologic diagnosis by retrograde ureteropyelography, such as vesicoureteral reflux (VUR) (Xia et al., 2012).

According to the recommendation by the FDA, the optimal dose of SonoVue^®^ in adult was 2.4 ml/time, and the dose range was 1.0–4.8 ml depending on the target organs and the equipment (Piscaglia et al., 2012). In the previous studies, many researchers started with different contrast agent administration strategies, based on the purposes of the study, various ages and weights of subjects, and the equipment used, especially the frequency of probes. The additional dose was necessary when performing CEUS in patient with large body masses, in a deep target organ or lesion, or using a high-frequency probe. In early period, it was suggested that 0.1 ml should be added for every additional year or additional kilogram; and the dose of 2.4 kg was suitable for the subject with a weight over 24 kg in Europe (Stenzel, 2013). There were studies with a fixed dose of 0.1, 0.5, 1.2, 2.4, and 4.8 ml reported (Bonini et al., 2007; Piskunowicz et al., 2012; Jacob et al., 2013; Knieling et al., 2016). In the recent guideline in USA, the recommended dose was 0.03 ml/kg and the maximal dose was 2.4 ml in children (Lumason prescribing information website). We used different administration strategies in our study. It was found that the wash-out time was associated with the dose. The larger dose had a longer wash-out time. For reducing the detention time, the dose of 0.03 ml/kg may be the better option than the dose of 2.4 ml/time on the basis of the same diagnostic efficacy.

In our study, there were three cases of mild allergic reactions, which occurred after administering CEUS from different batches. The earliest allergy occurred within 5 min after injection of contrast agent, and relieved gradually after the immediate administration of dexamethasone injection. For the other allergic reactions, we used methylprednisolone dexamethasone, and acquired a better therapeutic effect. By now, it was known that methylprednisolone had a quicker effectiveness than dexamethasone for anti-allergy therapy. And what’s more, some researchers suggested that, using dexamethasone for prevention should be a routine before CEUS examinations (Sidhu et al., 2018). However, for the sake of safety, we didn’t take that measure in our study. Instead it was stressed that emergency preparations needed to be in place for immediate response against such adverse effects. Besides the rash, there were three cases of hypotension which happened after administration of CEUS, which was completely resolved after anti-shock therapy. Above all, intravenous CEUS with SonoVue^®^ was generally safe.

According to our experience from the trial, we made a conclusion for the measures dealing with abrupt adverse effects. Firstly, to stop administration and to keep a suitable position—that is, lying down and raising legs when hypotension happened. Secondly, to keep the airways open and to provide oxygen should be done. And then the anti-allergy drugs should be intravenously injected, and the vital signs should be monitored in real time. For the anti-allergy therapy, we can use a dose of 1–2 mg/kg of methylprednisolone for the subjects with rash, and use a dose of 0.01 mg/kg of 0.1% adrenaline for the subjects with hypotension. And they can be used repeatedly 5–15 min after the first use if necessary. The hemodynamic change can be monitored by the arterial cannulation as well.

## Conclusion

Intravenous CEUS for pediatric applications was generally safe as observed with a low frequency of adverse effects. In addition, it was associated with better diagnostic effectiveness so that it can be performed for many kinds of medical issues.

## Limitation

There were limitations in our study. The study was in a single center, and lack of cooperation with other institutions. Besides, the disease entities were so complex that there was no specific CEUS modality. In the next step, we will collaborate with other institutions and make our study more standard and systematic, which are favorable for promoting the CEUS application in children and make it more feasible.

## Data Availability Statement

The datasets generated for this study are available on request to the corresponding author.

## Ethics Statement

The studies involving human participants were reviewed and approved by the Chinese Ethics Committee (No: 2016[003], ChiCTR-ONh-16009236). Written informed consent to participate in this study was provided by the participants’ legal guardian/next of kin. Written informed consent was obtained from the individual(s), and minor(s)’ legal guardian/next of kin, for the publication of any potentially identifiable images or data included in the article.

## Author Contributions

The corresponding author BX conceived and conducted the study and gave the guidance in the whole study. The first author M-yM collected and chose the cases, performed the literature search and data extraction, and wrote the paper. The author W-IC assisted to the statistical analysis. Other authors participated in the study and provided some professional guidance. All authors read and approved the manuscript.

## Conflict of Interest

The authors declare that the research was conducted in the absence of any commercial or financial relationships that could be construed as a potential conflict of interest.
